# Introducing a new routing method in the MANET using the symbionts search algorithm

**DOI:** 10.1371/journal.pone.0290091

**Published:** 2023-08-25

**Authors:** Shayesteh Tabatabaei

**Affiliations:** Department of Computer Engineering, Higher Education Complex of Saravan, Saravan, Iran; Khon Kaen University, THAILAND

## Abstract

A wireless MANET network is a grouping of wireless nodes that communicate without the use of a centralized network infrastructure. The lack of reliable elements, such as routers, as well as severe resource constraints, have an important impact on the performance of these networks. To improve the efficiency of MANET, intelligent routing algorithms are required; in fact, the design of a smart and efficient routing algorithm can significantly affect the efficiency of these networks. In this regard, this paper proposes an intelligent routing method for MANET networks based on (SOS) symbiotic organism search. The proposed method is implemented in dynamic environments by considering four important criteria: available bandwidth, remaining battery energy, mobility speed, and hop count. The simulation results show that the learning process of the symbionts search algorithm has a significant impact on network performance and outperforms the FBRP algorithms in terms of throughput rate, data loss rate, packet delivery rate, and the number of hops.

## 1. Introduction

Because of the growing popularity of mobile devices and wireless networks in recent years, the MANET network has become one of the most animated and dynamic fields of communication. MANET is essentially an organization-less network of transportable devices with wireless communication capabilities that can dynamically connect at any time and location. These networks are made up of portable devices such as laptops, smartphones, sensors, and so on that communicate with one another via wireless links [1]. These devices work together to provide essential network functions without an immovable organization in a distributed manner.

MANET networks allow devices to move freely and autonomously in any direction. The nodes can detach and reconnect to the network at will. As a result, variations in the node’s link state with other nodes are experienced regularly. The movement in the MANET network changes link states and other wireless transmission characteristics such as attenuation, multipath propagation, interference, and so on. This issue poses challenges for routing protocols operating in MANET.

Furthermore, the power of MANET nodes for communication is provided by a limited-capacity battery. This battery may run out of power and turn off during data routing. This issue invalidates the route between source and destination, so optimal energy consumption in this type of network is essential [2].

As a result, in order to prevent reducing the quality of services in such a network (whose topology is constantly changing and whose nodes have energy constraints), it is necessary to design intelligent routing protocols that minimize energy consumption and provide stable routes between sources and destinations.

Achieving an optimal routing algorithm in MANETs is an NP-hard problem that can be solved by soft computing and optimization techniques [3]. Based on this, in recent years, many smart algorithms have been presented for routing mobile ad hoc networks, with different aims, among which FBRP (to reduce energy consumption) can be mentioned.

In MANETs, other routing criteria, such as available bandwidth and mobility speed should also be checked to improve the quality of the link and the performance of the routing protocols. Each criterion can be viewed as a collection of measurements that helps the routing algorithms to estimate the new weight for each step/link in the intended routes. Although so far, various routing criteria have been proposed to evaluate link quality, only a few of them have been implemented and tested in a real network simultaneously [4]. Not paying attention to any of these criteria can reduce the efficiency of the network; thus, considering a limited number of them in routing can be a significant weakness for such protocols.

As said above, changes in the network topology are one of the prominent features of the MANETs, so always establishing a stable path between source and destination in this type of network is a major challenge; therefore, routing protocols should be created to discover routes with high reliability until the end of the data transmission phase.

In this study, a new method for MANET routing using the symbiotic organisms search algorithm is proposed.

The symbiotic organisms search algorithm has three separate and strong processes: mutualism, communication, and parasitic life. The proposed method improves the solutions in the population with the help of these three processes. One of the advantages of this algorithm is not having any parameters to adjust the two operations between exploration and productivity. Therefore, the processing cost is expected to be reduced due to the simplicity of implementing the algorithm. This issue decreases the energy consumption in MANET.

One of the important features of the SOSBRA algorithm is that it encourages nodes to thoroughly investigate their surroundings before converging on the best global route. As a result, it prevents falling into the local optimum. Therefore, paths with high stability are discovered.

To evaluate the proposed method, in addition to this method, the FBRP algorithm (as a well-known intelligent protocol) is also simulated in OPNET software.

An important point that needs to be mentioned is that: the proposed method is implemented in dynamic environments by considering four important criteria of available bandwidth, remaining battery energy, mobility speed, and the number of hops. This issue is considered an important strength of this algorithm, and it will guarantee the achievement of reliable routes in different time and place conditions. However, other research works have yet to consider these four parameters simultaneously.

This paper’s main contribution is the use of the Modified routing algorithm to enhance the performance of mobile ad hoc networks as follows:

The hello message is used to identify the new neighbor nodes, and the neighbor table is updated as new responses are received from the neighbor node.

The proposed method is implemented in dynamic environments.

We consider available bandwidth, remaining battery energy, mobility speed, and hop count to choose the best path.

We employ a sophisticated routing technique based on (SOS) symbiotic organism search, which has been shown to perform well and achieve high convergence accuracy when optimizing multidimensional functions with higher dimensions.

The proposed routing algorithms implement over mobile ad hoc nodes using a network simulator (OPNET).

This article is divided into the following sections: The second section describes related work on routing protocols in MANETs, the third section states the proposed method, and the fourth section provides simulation and simulation results in the OPNET simulator. The conclusion is stated in the fifth section.

## 2. Related works

Omar et al. [5] presented a protocol based on source routing, in which the source node inserts routing information into data packets and allows the intermediate nodes to find the route to the destination. In the proposed method, instead of complete information about the address of the intermediate nodes, the data packets carry only a small numerical value that is a summary of the route to the destination and helps the data packets find their way to the destination.

The proposed method is divided into route discovery and data packet routing phases. If a source node’s cache does not contain any routes to its destination, the route discovery phase is started by using route request packets (RREQ) before sending data. After discovering the route, the data is sent from the source to the destination. In this article, only the mobility criterion was used for routing, and the critical criterion of battery energy level was not considered.

Khalid et al. [6] introduced a response rate-aware routing protocol for MANET. In the proposed method, nodes check the ongoing transmissions to estimate the transmission rate. In addition, instead of updating the entities in the routing table, a uniform rate of Hello messages is sent to the sending node. If the sending node receives that message, it will know that it has the correct information about the status of the sending rate. This node then stores this information and sends it to all of the nodes from which the data is received. Compared to other algorithms, this algorithm finds reliable routes while reducing the overhead caused by sending Hello messages periodically.

In the article [6], only two criteria, Medium Time Metric, and the hop count were used. This paper did not take into account the amount of energy consumption, the mobility of nodes, and the available bandwidth for routing, which are among the basic and determining parameters in MANET routing protocols.

Sumathi and Priyadharshini [7] introduced a new routing algorithm that uses multiple channels to improve the efficiency of MANETs. The proposed interlayer method employs a channel allocation strategy and collision control techniques. Channels are divided into two categories: data channels and control channels.

The data channel is used for data transmission, while the control channel is used for scheduling. The authors of this article assume that each node has a wireless network card and can send or receive data on any channel at any time. In the article, only the packet capacity criterion was checked, and the other important criteria were not mentioned.

Wang and Garcia [8] presented a new interlayer routing protocol that uses the multi-factor Q-learning algorithm to control transmission rate and node energy. This method assigns a reward to the next steps by continuously observing the reward of all status-actions, then uses the links with the highest reward for each route leading to the destination. The proposed algorithm reduces the amount of energy consumed by mobile nodes.

In this article, the criteria of node mobility speed and available bandwidth of the node were not checked.

Tabatabaei and Behravesh [9] used the reinforcement learning method to find a highly reliable route in MANETs. To calculate the reward, this method uses fuzzy logic with three inputs: the available bandwidth, the number of steps, and the remaining energy of the battery. In this article, mobile MANET routing was done once with fuzzy logic, once with reinforcement learning, and finally with a combination of these two algorithms. Simulations showed that the combined method produced a better result regarding energy consumption, efficiency rate, and latency. In this article, the mobility criterion (which changes the topology with the nodes’ mobility) was not considered.

Chettibi and Chikhi [10] proposed an Ad hoc On-Demand Distance Vector (AODV) routing protocol based on the fuzzy method to reduce energy consumption in MANETs. This algorithm makes the decision about sending the route request using a Mamdani fuzzy logic system with the inputs, the remaining battery energy, and the amount of energy loss of mobile nodes.

In this article, only the energy level of the battery was considered, and other important criteria, such as mobility and available bandwidth, were not considered.

Chatterjee and Das [11] used the ant colony optimization algorithm to improve the dynamic source routing protocol (DSR). Routing in the proposed method is done using a technique similar to the biological behavior of ants to find food, where ants first scatter in all directions. This method discovers multiple routes and has a high packet delivery rate, low end-to-end delay, and low energy consumption.

In this article, only three criteria, length, congestion, and reliability of the path, were used for choosing the paths; and the important criteria, such as the level of battery energy and mobility, were not considered.

In [12], the best routes were chosen using an interlayer routing method and a fuzzy method considering decision criteria such as node mobility and energy parameters. This algorithm allows mobile nodes to find better routes with more bandwidth and higher quality of service. This method also extends the network lifetime and improves multimedia flow quality.

Unfortunately, this article did not consider important criteria such as available bandwidth, distance to the destination, or the number of hops.

In [13], a Fuzzy logic-based on-demand routing protocol (in brief FBRP) for MANETs was proposed that selects routes based on battery energy level and mobile node speed. The simulation results showed that the proposed method has good performance and high error tolerance, especially during node failure.

It should be noted that in this article, only two criteria of battery energy level and mobility were considered for routing.

In 2019, to evaluate the fit of each node in WSNs, a clustering method-based optimized invasive weed algorithm using fuzzy modeling was proposed [14]. This method can choose the best node to be the cluster head and extend the network’s lifespan. The simulation results showed that it could reduce the number of dead nodes in each run while lowering sensor energy consumption.

Although the proposed method can quickly find the global optimal using the optimized aggressive weed algorithm, it suffers from a high processing cost (due to using fuzzy logic).

Gorgich and Tabatabaei [15] presented a new method for improving energy consumption in wireless sensor networks using a fish swarm optimization algorithm. To evaluate the proposed protocol, the protocol was compared with the ERA (Energy Aware Routing Algorithm) protocol. The disadvantage of this method is that fish optimization is used in static environments, and the convergence speed may be reduced in dynamic environments such as sensor networks.

In [16], a fuzzy inference system used the shuffled frog leaping algorithm to automatically configure and optimize the base rule table. This method has two steps: cluster head selection (CH) and parent selection. The CHs are chosen from the candidate nodes based on the fuzzy output, energy threshold, and the overlap rate of adjacent CHs. The parent selection phase starts with determining the network’s CHs level. The parents of each CH are determined at the end of this step. Finally, the information gathered by the CHs is forwarded to the BS by their parents. Although this method saves energy, it has a high processing cost.

In [17], an improved protocol based on AODV was proposed that works based on clustering in VANET. This protocol uses fuzzy logic to select reliable routes between cluster members. Fuzzy logic input parameters are the link expiration time and link reliability. Also, in this article, Tabu search was used for routing between the cluster heads and the sinks based on criteria distance, direction, and velocity. The simulation results indicated the suitable performance of the proposed protocol in terms of the average packet delivery rate, the average end-to-end delays, and the number of packet losses. In this method, the energy level of the battery was not considered.

In [18], an inter-vehicle routing protocol using fuzzy logic was proposed to select the most reliable link and to avoid packet replays in the geographic range. This method uses effective distance, direction, and velocity parameters to find the next-hop node. The simulation results show that the proposed protocol increases packet delivery rate and decreases packet loss rate and end-to-end delay.

In this article, only two parameters, the link expiration time and the probability adapt density, were considered. The proposed method had a high processing cost.

In [19], the division of the dedicated short-range communication channel with three types of priority was used to transfer the data message quickly. Furthermore, the multi-hop directional routing method was used to increase reliability. Moreover, the storage and distribution method was used during communication gaps to increase efficiency. The processing cost was high in the proposed method since several methods were combined.

In [19], a fuzzy logic-based routing method with authentication capability in VANET was presented. This method includes the clustering, routing, and authentication phases. In the clustering phase, vehicles are clustered. Data routing is done in the routing phase. In the authentication phase for secure data, the packets use an authentication mechanism based on message authentication code and symmetric key cryptography. The simulations showed that the proposed method improves end-to-end delay, packet collision, packet delivery rate, packet loss rate, and throughput. However, it increases the routing overhead slightly.

According to what has been said above, the common routing protocols in MANETs consider only one or two of four important parameters: available bandwidth, remaining battery energy, mobility speed, and the number of hops to discover the best routes.

But not paying attention to any of these parameters in different times or place situations can prevent finding the local optimum and discovering reliable routes.

As a result, Implementing a comprehensive protocol that considers all the above four parameters in its evaluation simultaneously is essential.

## 3. Proposed method

### 3-1 Introduction

A MANET is a network of battery-powered mobile nodes with limited bandwidth. One of the most fundamental challenges in this type of network is routing. An intelligent routing protocol based on symbiont search algorithms is presented in this study. Cheng and Prayogo [20] are the pioneers of the symbiont search algorithm.

This algorithm is a population search method that simulates how living things interact with each other to survive in nature. The symbiont search algorithm begins with a population known as an ecosystem. This algorithm searches the answer space of the problem for the best answers using the ecosystem and its mechanism. The ecosystem comprises a number of organisms or living things, each representing a candidate to answer the problem. In fact, every organism is a point with the number of components corresponding to the number of problem decision variables. As a result, the value of the objective function for each organism can be calculated. The value of the obtained objective function indicates the organism’s compatibility with nature. The task of generating a new population in the symbiotic organism search algorithm is the responsibility of three actors, each representing a type of symbiosis including mutualism, commensalism, and parasitism.

The most important symbiotic relationships between creatures in nature are:

- Mutualism symbiosis (a relationship between two different species that benefits both),
- Commensalism symbiosis (a relationship between two different species that benefits one but has no effect on the other), and
- Parasitic symbiosis (a symbiosis between two different species that is beneficial for one and harmful for the other.).

Thus, this search method, which is inspired by the pattern of symbiosis between living things in nature, employs three stages: mutualism symbiosis, commensalism symbiosis, and parasitic symbiosis. Each phase of this algorithm is described below:

- Phase one: mutualism symbiosis (win, win)

At this phase, one answer X_j_ (i ≠ j) is chosen randomly from the population for each answer X_i_ in the population. Then, to improve the fit, both X_i_ and X_j_ answers enter into a mutualism symbiosis relationship. X_i_ and X_j_ are produced according to Eqs (1) to (3).


$${\text{X}}_{\text{inew}}{=\text{X}}_{\text{i}}+\text{rand}(0,1)\times {(\text{X}}_{\text{best}}-{\text{mutual}}_{\text{vector}}\times {\text{BF}}_{\text{1}})$$ (1)

$${\text{X}}_{\text{jnew}}{=\text{X}}_{\text{j}}+\text{rand}(0,1)\times {(\text{X}}_{\text{best}}-{\text{mutual}}_{\text{vector}}\times {\text{BF}}_{\text{2}})$$ (2)

$${\text{mutual}}_{\text{vector}}=\frac{{\text{X}}_{\text{i}}+{\text{X}}_{\text{j}}}{\text{2}}\;$$ (3)

In the above relationships, rand (0,1) is a random number in the range [0,1], and X_best_ represents the population’s best response. BF_1_ and BF_2_ are two coefficients that can be either 0 or 1. In fact, these coefficients demonstrate that the degree of the benefit provided by two beings to each other in a symbiotic relationship is not always equal; and it can even be many times more for one of the two creatures than the other. This algorithm considers this amount to be a maximum of two times. If the fit of the answers X_inew_ and X_jnew_ be better than the fit of the answers X_i_ and X_j_, these two new answers replace the previous values; otherwise, the previous answers will be preserved.

- Phase two: commensalism or self-help symbiosis (win—indifferent)

At this stage, one answer X_j_ (i ≠ j) is randomly selected from the population for each answer X_i_ in the population. Then, X_i_ and X_j_ form a self-help symbiotic relationship to improve the fit of answers. The new candidate’s answer X_inew_ is generated by Eq (4).


$${\text{X}}_{\text{inew}}{=\text{X}}_{\text{i}}+\text{rand}(-1,1)\times {(\text{X}}_{\text{best}}-{\text{X}}_{\text{j}})$$ (4)

The reason for selecting the interval [-1,1] to generate a random number in this equation is to increase the scanning power of the search for symbionts. After using this step, If the fit of the answer X_inew_ is better than the fit of the answer X_i_, X_inew_ replaces X_i_; Otherwise, X_i_ will be preserved.

- Phase three: parasitism symbiosis (win-lose)

At this phase, one answer X_j_ (i ≠ j), is chosen randomly for each answer X_i_ in the population. The answer X_k_ is then generated by applying a random change to X_i_; if the fit of X_k_ is better than X_j_, it replaces X_j_, and otherwise, X_k_ will be disappeared.

The most significant advantage of symbiont organisms is that they lack a specific parameter that can reduce the number of calculations (except the population parameter, which is also present in other metaheuristic methods, no other parameter is seen in this algorithm).

The following section describes how to use this algorithm to route a MANET.

### 3-2 Determining the best route by the symbionts search algorithm

In this stage, the source node creates a special packet called the route request packet to know the next hop to the destination. The source node then sends the request packet to neighbors as a multicast to find the location of the destination. Before sending the packet, the number of hops is reduced to zero. When a route request packet arrives at an intermediate node, the attribute pairs of the source address and the request ID are first checked in a local table that stores records of such packets to see if it has already been received and processed. If it is a duplicate, the packet will be deleted, and its processing will be terminated. If it is not a duplicate, these attribute pairs are entered in the record table to prevent a similar package from being processed in the future, and the processing process continues. The receiver then searches its routing table to find the destination address. If a new route to this destination is discovered, a route reply packet is returned to the source so that the source knows how to reach the destination. If the receiver does not have a new route to the destination, it increments the value of the hop counter by one unit and republishes the route reply package around it.

This process is repeated until the route request message arrives at the destination. The destination node generates a route reply packet in response to the incoming request. The above node copies the source and destination address fields (extracted directly from the route request packet) to the route reply packet. This packet is sent only to the node through which the route request packet was received. A unit is added to the value of the hop counter field in each node so that each node that sees it knows how far it is from the destination node.

Each intermediate node for each destination in the routing table checks the existence of the candidate node for the next hop. If there are at least two candidate answers for a destination, that destination is considered an element of the candidate answer in the last node. On the other hand, only nodes can implement the ultra-innovative symbionts search algorithm if the candidate answer contains at least one element. As a result, the candidate answer of a node may have three elements, while the candidate answer of the neighboring node may have only one element. Assume k is the set of candidate answers and, is the answer i_th_ of the candidate.

Also, suppose that the vectors X_j_, Y_j_ and Z_j_ are respectively the locations of the solution of element K_i_, on the X, Y and Z axes, so that *X*_*ij*_, *Y*_*ij*_ and *Z*_*ij*_ are the locations of the selected next hop to the destination for the j_th_ of the destination node.

Now, each node that has the candidate answers chooses m random answers, where m is the size of the ecosystem’s population.

In short, and to summarize the stated content, it can be said that:

In the route discovery phase, the route request packet that is generated and sent by the source will be received by intermediate nodes. For each intermediate node, there is a table called the routing table, which contains information on the available routes from that intermediate node to the specified destination. For the specified destination in the route request packet, the intermediate node checks the existence of the candidate node for the next hop from its routing table. Nodes having the candidate answer add a unit to the variable routing_num_iteration and set the variable I to 1. Then, these nodes send the route request message containing the IP of the candidate node to its neighbors (as the next step for the destinations in the routing table) in a multicast manner.

Then the intermediate node sends a useful message containing four parameters of available bandwidth, mobility speed, number of hops, and the energy level of the neighboring node to the next hop node to the destination so that the next hop node calculates the fitness of the best node.

Assume there is a threshold value for each of the four criteria. The node will receive more points if the criterion value exceeds the threshold. For example, the battery energy level is 100, and the threshold is 50. If a node’s battery energy is 60, its fit score is 0.6, and this process for each criterion is repeated. The fitness of the number of hops is calculated in Eq (7), and the fitness of the node’s mobility speed is calculated in Eq (8).

$$f_{p}(K)=\frac{f_{PR}(K)}{Max_{p}}$$ (5)

Where f_P_(K) is the function of energy fitness, and f_PR_(K) is the amount of remaining energy. MAX_p_ is the maximum energy level of the battery.

$$f_{BW}(K)=\frac{f_{ABW}(K)}{Max_{BW}}$$ (6)

Where f_ABW_(K) is the fit function of bandwidth, ABW is the amount of available bandwidth, and MAX_BW_ is the maximum bandwidth level.

$${\text{f}}_{\text{D}}(\text{K})=1-\frac{{\text{f}}_{\text{H}}(\text{K})}{{\text{Max}}_{\text{D}}}$$ (7)

Where f_D_(K) represents the fit function for the distance between nodes, H represents the number of hops, and MAX_D_ represents the maximum distance.

$$f_{S}(K)=1-\frac{f_{SN}(K)}{Max_{S}}$$ (8)

Where f_S_(K) is fit function of the mobility speed, f_SN_(K) is the node mobility rate, and MAX_S_ is the maximum mobility speed of the nodes.


$$f_{Total}(K)=f_{BW}(K)+f_{D}(K)+f_{S}(K)+f_{p}(K)$$ (9)

The usefulness of each intermediate node’s candidate answer is then calculated using Eq (9), and the best candidate answer is identified and placed in k_best_. Then, for each candidate answer k_*i*_, the vectors X_*i*_، Y_*i*_ and Z_*i*_ are calculated, and finally, the following steps are performed:

#### Two-way utility stage

In this step, for each intermediate node (current candidate node) k_*i*_, a candidate node named k_j_ (i ≠ j) (new candidate node) is randomly selected.

Then, to improve the fit of both answers (current and new), the vectors X_*i*_، Y_*i*_ and Z_*i*_ form a symbiotic relationship. The new candidate answers for X_*i*_، Y_*i*_ and Z are generated as a result of the mutual symbiosis between the two responses using Eqs (10) to (18).


$${\text{X}}_{\text{inew}}{=\text{X}}_{\text{i}}+\text{rand}(0,1)\times {(\text{X}}_{\text{best}}-{\text{mutual}}_{\text{vectorXij}}\times {\text{BF}}_{\text{1}})$$ (10)

$${\text{X}}_{\text{jnew}}{=\text{X}}_{\text{j}}+\text{rand}(0,1)\times {(\text{X}}_{\text{best}}-{\text{mutual}}_{\text{vectorXij}}\times {\text{BF}}_{\text{2}})$$ (11)

$${\text{Y}}_{\text{inew}}{=\text{Y}}_{\text{i}}+\text{rand}(0,1)\times {(\text{Y}}_{\text{best}}-{\text{mutual}}_{\text{vectorYij}}\times {\text{BF}}_{\text{1}})$$ (12)

$${\text{Y}}_{\text{jnew}}{=\text{Y}}_{\text{i}}+\text{rand}(0,1)\times {(\text{Y}}_{\text{best}}-{\text{mutual}}_{\text{vectorYij}}\times {\text{BF}}_{\text{2}}^{})$$ (13)

$$Z_{\text{inew}}{=\text{Z}}_{\text{i}}+\text{rand}(0,1)\times {(\text{Z}}_{\text{best}}-{\text{mutual}}_{\text{vectorZij}}\times {\text{BF}}_{\text{1}})$$ (14)

$$Z_{\text{jnew}}{=\text{Z}}_{\text{j}}+\text{rand}(0,1)\times {(\text{Z}}_{\text{best}}-{\text{mutual}}_{\text{vectorZij}}\times {\text{BF}}_{\text{2}})$$ (15)

$${\text{mutual}}_{\text{vectorXij}}=\frac{X_{i}+X_{j}}{2}$$ (16)

$${\text{mutual}}_{\text{vectorYij}}=\frac{Y_{i}+Y_{j}}{2}$$ (17)

$${\text{mutual}}_{\text{vectorZij}}=\frac{Z_{i}+Z_{j}}{2}$$ (18)

The nearest neighboring node to X_inew_, Y_inew_ and Z_inew_ is considered the candidate answer for K_i_. Also, the nearest neighboring node to X_jnew_, Y_jnew_ and Z_jnew_ is considered the candidate answer for K_j_. The usefulness of K_inew_ and K_jnew_ is then calculated using an Eq (9).

If the usefulness of candidate answers K_inew_ and K_jnew_ is greater than that of K_i_ and K_j_ candidate answers, respectively, K_inew_ and K_jnew_ will replace K_i_ and K_j_.

#### One-way utility stage

At this step, one response K_j_ (i ≠ j) is randomly chosen from the population for each response k_*i*_. The vectors X_*i*_، Y_*i*_ and Z_*i*_ are then Calculated; these vectors enter a commensalism symbiotic relationship. Eqs (4), (19) and (20) are used to generate new candidate answers for X_*i*_، Y_*i*_ and Z_*i*_.


$${\text{Y}}_{\text{jnew}}{=\text{Y}}_{\text{j}}+\text{rand}(-1,1)\times {(\text{Y}}_{\text{best}}-{\text{Y}}_{\text{j}})$$ (19)

$${\text{Z}}_{\text{inew}}{=\text{Z}}_{\text{i}}+\text{rand}(-1,1)\times {(\text{Z}}_{\text{best}}-{\text{Z}}_{\text{j}})$$ (20)

The nearest neighbor node to X_inew_, Y_inew_ and Z_inew_ is considered as the candidate answer K_inew_. Eq (9) is used to calculate the usefulness of the candidate answer K_inew_. If the usefulness of K_inew_ is greater than that of K_i_, K_inew_ will replace K_i_.

#### Parasitism stage

In this step, for the IP of each candidate node k_i_ in the route request packet, k_j_ (i ≠ j) is randomly selected from among the new candidate nodes. The corresponding vectors X_*i*_، Y_*i*_ and Z_*i*_ are then obtained.

The worst candidate answer K_Worst_ is Identified from the set of candidate answers. Using the location of nodes K_j_, K_Worst_ and K_best_ on the X, Y and Z axes, the approximation position of the parasitic vector K_parasitic_ is calculated (using Eqs (21)–(23).

The nearest neighbor node to X_parasite_, Y_parasite_, and Z_parasite_ is considered the candidate answer K_parasitic_.

If the usefulness of K_parasite_ is greater than the usefulness of K_j_, K_parasite_ replaces K_j_.


$${\text{X}}_{\text{parasite}}{=\text{X}}_{\text{i}}+\text{rand}(0,1)\times {(\text{X}}_{\text{best}}-{\text{X}}_{\text{worst}})$$ (21)

$${\text{Y}}_{\text{parasite}}{=\text{Y}}_{\text{i}}+\text{rand}(0,1)\times {(\text{Y}}_{\text{best}}-{\text{Y}}_{\text{worst}})$$ (22)

$${\text{Z}}_{\text{parasite}}{=\text{Z}}_{\text{i}}+\text{rand}(0,1)\times {(\text{Z}}_{\text{best}}-{\text{Z}}_{\text{worst}})$$ (23)

The above steps are repeated until the termination condition is reached (the current answer becomes better than the previous answer) so that the best route to the destination can be found.

**Fig 1:**
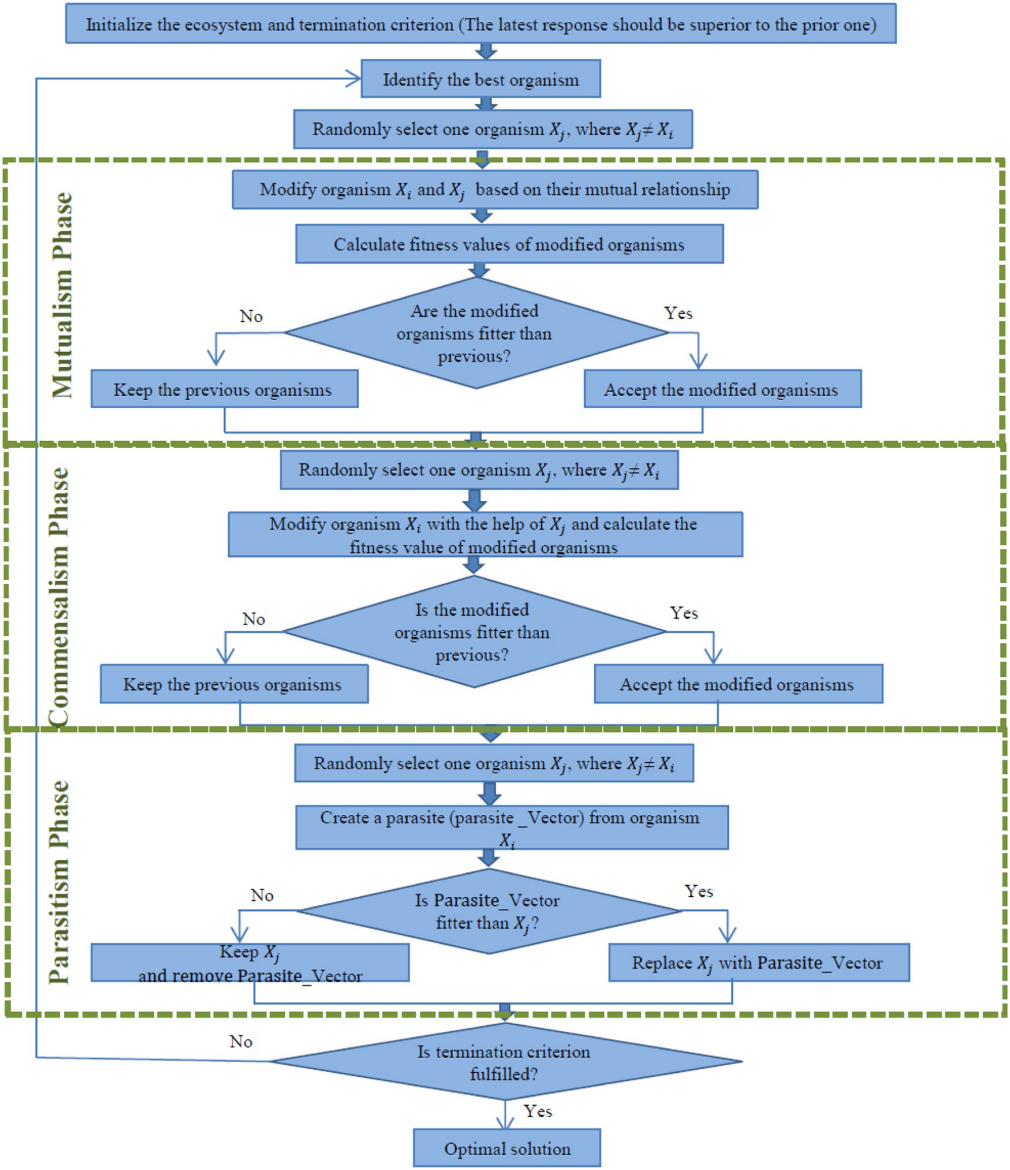
The general procedure of the proposed algorithm.

The general procedure of the proposed algorithm is shown in Fig 1.

By using such method, it is expected that the SOSBRA algorithm shows that a suitable efficiency (as depicted in Figs 4–7) due to advantages such as the following:

First, by moving towards the best global path, the SOSBRA algorithm prevents premature convergence. Second, it encourages nodes to thoroughly investigate their surroundings before converging on the best global route. Furthermore, as stated, the proposed method is implemented by considering four important criteria: available bandwidth, remaining battery energy, mobility speed, and the number of hops simultaneously.

## 4. Simulation of the proposed method

### 4-1 Simulation environment

**Fig 2:**
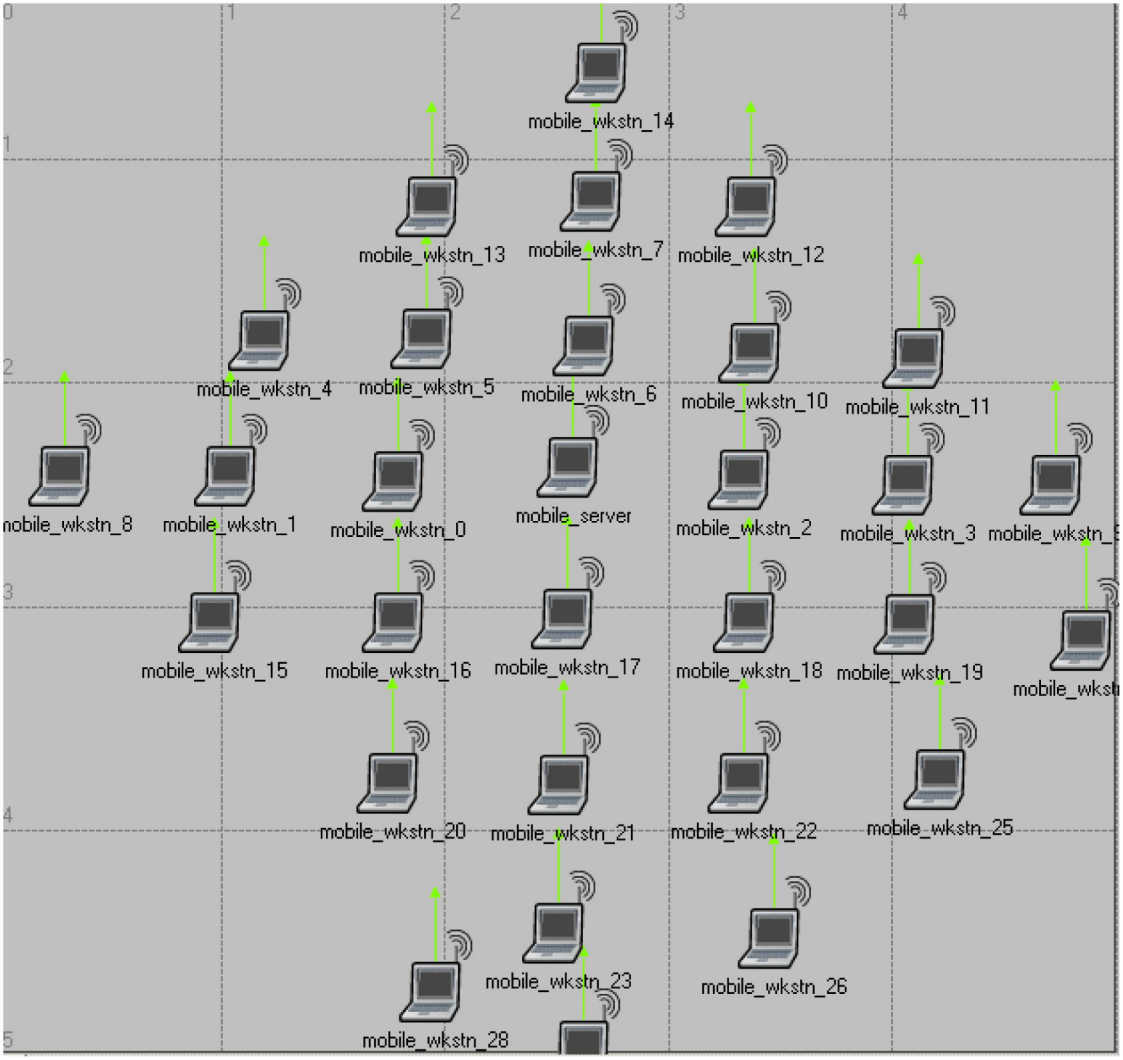
The network topology for 30 simulated mobile nodes.

In this paper, the proposed method is simulated and compared with the FBRP protocol using OPNET modeler simulation software. Table 1 shows the parameters of the implemented model for each layer, and Table 2 shows the simulation parameters. Fig 2 depicts the proposed network connection method, which considers 50 nodes. In the first scenario, mobile nodes are randomly distributed in the environment and are routed using the fuzzy method (FBRP) described in the article [13]. In the second scenario, nodes are randomly distributed and routed using the Symbiotic Organism Search Based Routing Algorithm (SOSBRA).

**Table 1:** Models that have been implemented for each layer.

Layer	Models
**Physical**	Free space
**Data Link**	802.11
**Network**	AODV
**Transport**	TCP
**Application**	FTP, HTTP

**Table 2:** Simulation parameters in the proposed protocol.

Simulation environment specifications	
**Number of mobile nodes**	30
**Simulation time**	500 second
**Simulated space**	100*1000 m^2^
**Transmission radius of each node**	250 m
**Number of calls to send data**	20 calls
**Number of data packets generated per call**	500 packets
**The size of each data packet**	1024 byte
**Rate of sending Packet on the network**	25 packet per second
**Bandwidth of each node**	2 MB per second
**Motion model**	Random Way Point
**Traffic model**	CBR
**Dissemination model**	Free space
**Interface access protocol**	IEEE802.11

**Fig 3:**
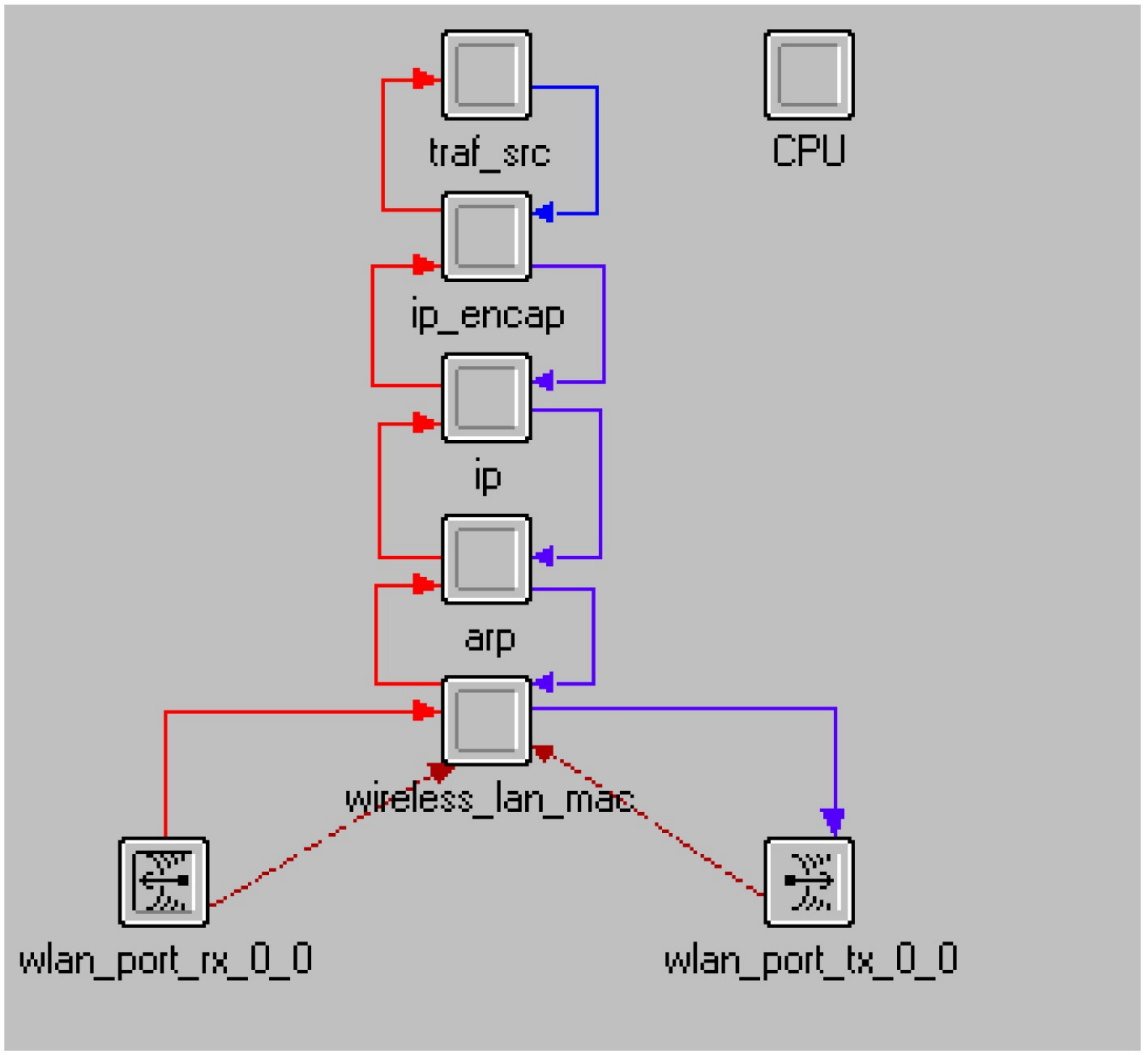
Model node editor.

The model node editor is shown in Fig 3.

Each node in a network is free to move independently in any direction, changing its connections to other devices frequently as a result. Each node also acts as a router by forwarding traffic unrelated to its own use.

### 4-2 Simulation results

The following criteria are used to evaluate the effectiveness of the proposed method.

- DR (delivery ratio): The ratio of the total number of messages delivered (m_del_) to the total number of messages generated (m_cre_) defines this parameter.
- Packet loss rate: The ratio of data packets lost at the destination to the packets generated at the source defines this parameter as a result of route failure or buffer overload.
- Throughput ratio: It is the number of bits transmitted in a unit of time between the source and destination.
- Route length: The number of steps / links that exist between the two points.

**Fig 4:**
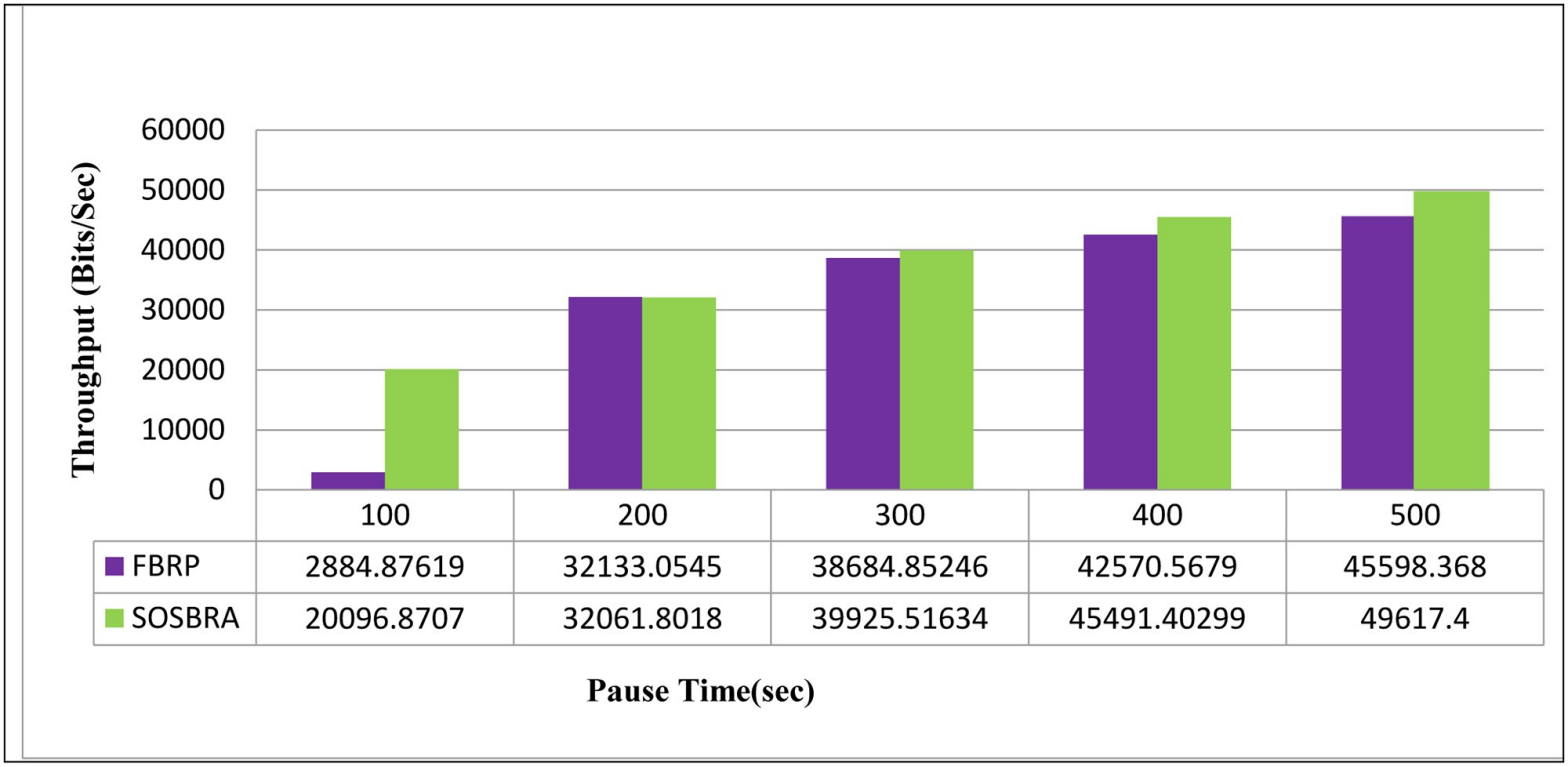
Throughput rate.

Fig 4 depicts the throughput rate. The horizontal axis represents simulation time, and the vertical axis represents the number of packets delivered at the time or throughput rate.

According to Fig 4, the ratio of the number of packets successfully delivered to the destination node to the total packets transmitted by the network nodes is lower in the FBRP protocol than the proposed SOSBRA method.

The most important feature of the SOSBRA algorithm is that it encourages nodes to thoroughly investigate their surroundings before converging on the best global route. As a result, it prevents falling into the local optimum. Therefore, paths with high stability are discovered. Furthermore, in the FBRP protocol, only two criteria are taken into account: mobility speed and node energy, while other important criteria, such as available bandwidth or distance to the destination, are not. On the other hand, the proposed method considers all four above parameters simultaneously; thus, the throughput rate is higher in this scenario.

**Fig 5:**
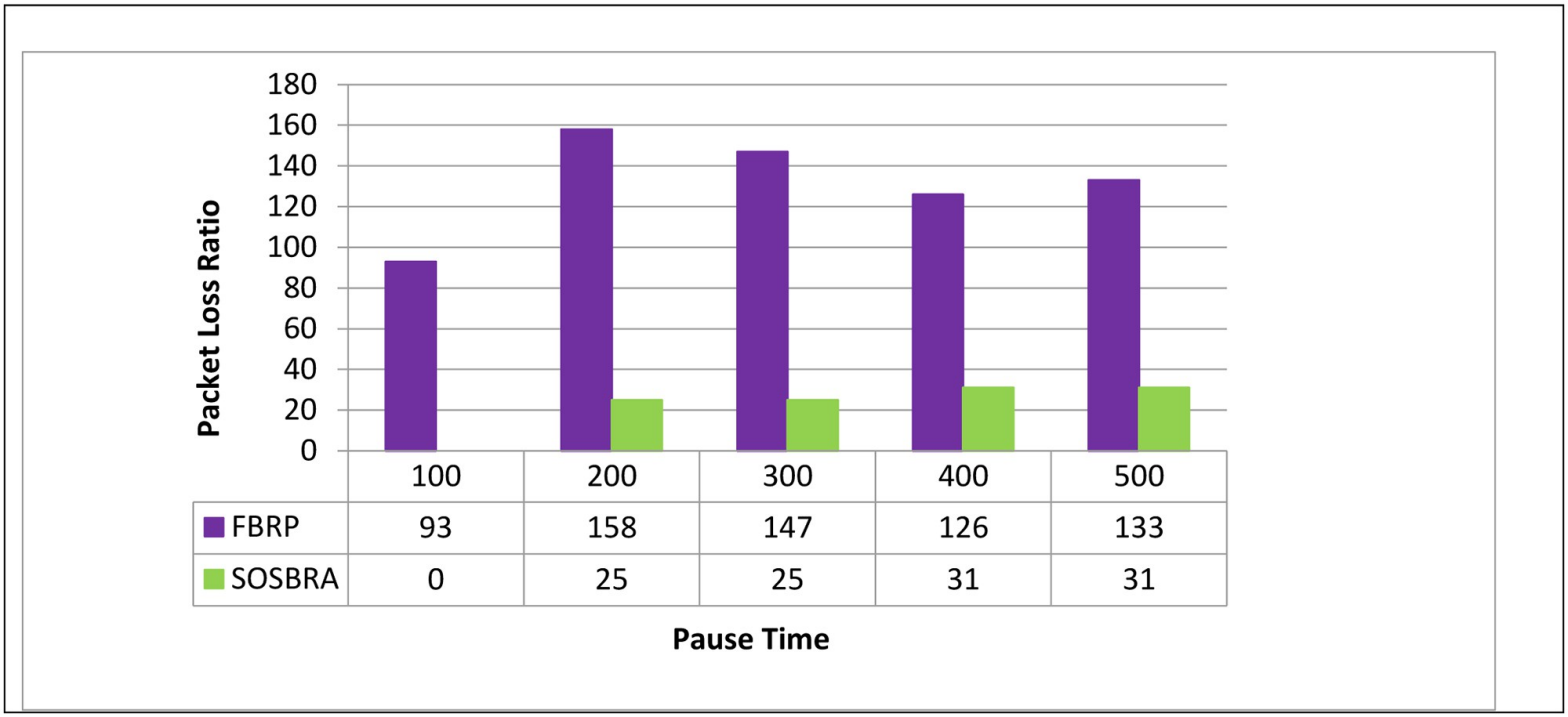
Data packet loss rate.

The data packet loss rate is depicted in Fig 5. The horizontal axis represents simulation time, while the vertical axis represents the data packet loss rate. Fig 5 shows that the FBRP protocol has a higher data packet loss rate than the SOSBRA method.

Since the lack of access to a route to the destination can be one of the reasons for the loss of data packets in the network, the SOSBRA method attempts to select routes that are stable, at least until the end of the data transfer phase. This issue reduces the number of lost data packets and the average delay.

It should be noted that the data loss rates in the FBRP protocol may also increase due to a lack of available bandwidth.

**Fig 6:**
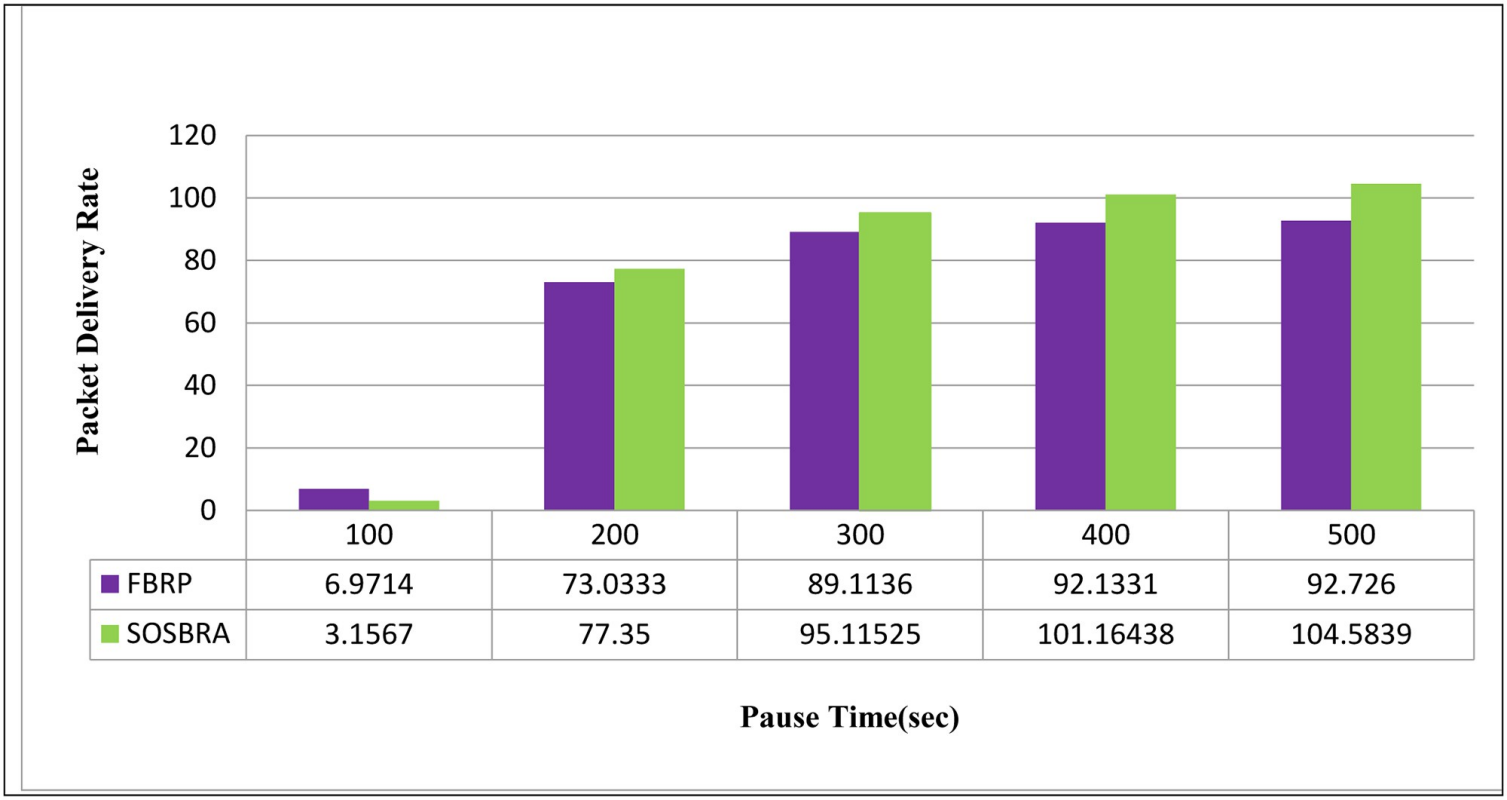
Data packet delivery rate at destination.

Fig 6 compares the data packet delivery rate of the proposed SOSBRA method with the FBRP protocol. The SOSBRA method will have a data packet delivery rate higher than the FBRP protocol because it finds a new route before failing caused by mobility or a lack of necessary bandwidth.

It should be noted that if the proposed method is not very successful in finding the route before failure, Since it prefers longer routes but more stable to the shortest unstable routes, It could take longer than FBRP.

According to Fig 6, the proposed method’s data packet delivery rate increases faster than the FBRP protocol as simulation time increases. Because the proposed method is capable of determining the most appropriate and stable links for data transmission, in other words, routes are created that will most likely lead the package to its destination.

**Fig 7:**
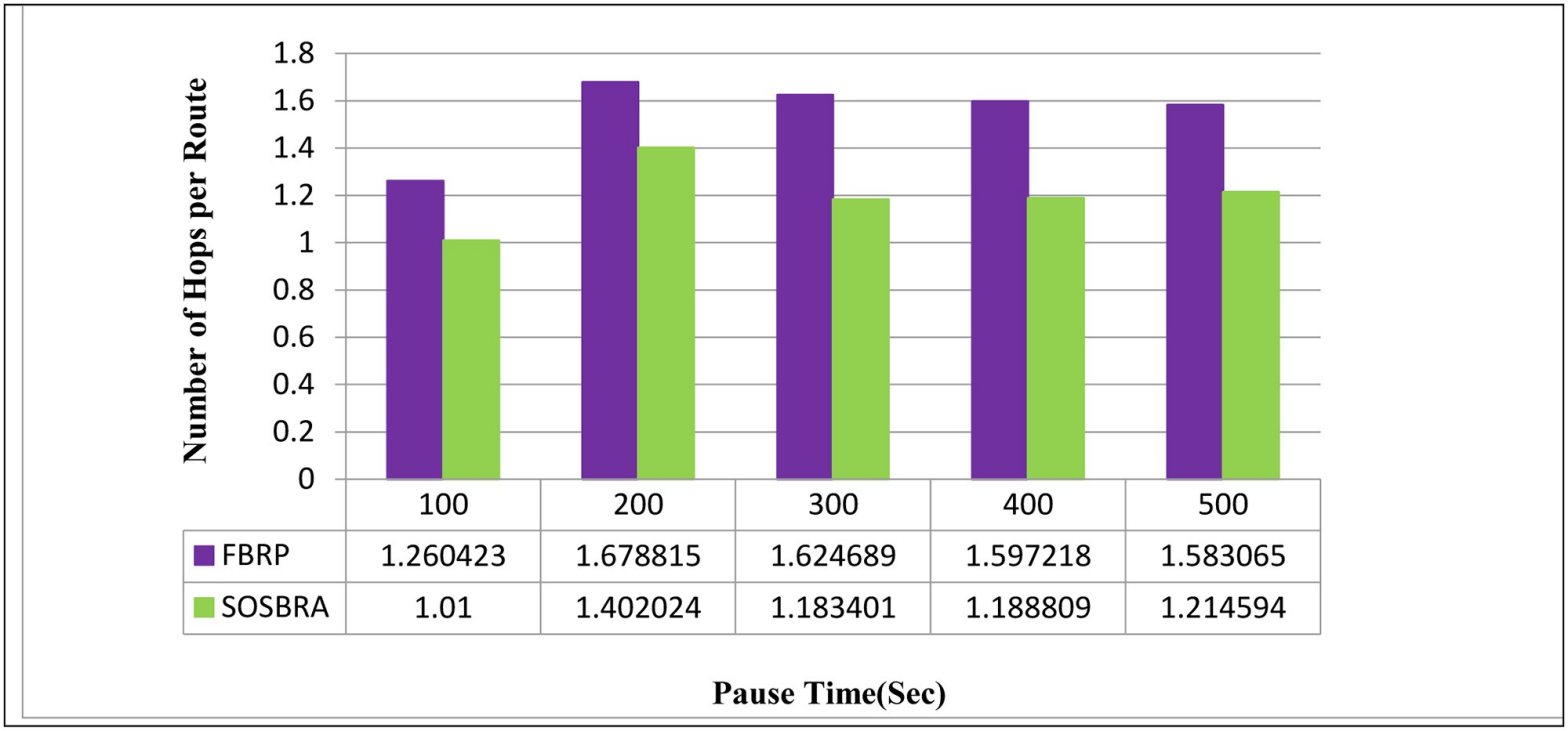
Number of hops to destination.

Fig 7 depicts the number of hops required to reach the destination using the proposed SOSBRA method and the FBRP protocol. The horizontal axis represents simulation time, while the vertical axis represents the number of hops. Fig 7 shows that the proposed protocol chooses a shorter route than the FBRP protocol. This problem originates from the fact that in the proposed protocol, the parameter of hops number is considered one of the four important criteria in routing. At the same time, route stability and distance issues are also taken into account. Furthermore, in the FBRP protocol, the route may be invalidated due to a lack of bandwidth.

## 5. Conclusion

In this article, considering that meta-heuristic algorithms inspired by nature are widely used to find the right answer in optimization problems, a new method using the search algorithm symbiotic organisms called SOSBRA has been proposed. The symbionts search algorithm is one of the most recent meta-heuristic algorithms that has demonstrated good performance and high convergence in optimizing multidimensional functions with higher dimensions. The proposed method can increase the data delivery rate by selecting routes with high stability between source and destination. In the OPNET simulator, SOSBRA and the FBRP protocols are simulated. Comparing the results shows that SOSBRA performs better for network features such as power consumption, throughput rate, number of hops, and latency. By moving toward the best global direction, the SOSBRA algorithm prevents premature convergence. Furthermore, it encourages nodes to thoroughly investigate their surroundings before converging on the best global route.

According to the high speed of MANETs or other factors such as; the rapid disconnection of routing links, the sudden shutdown of nodes due to battery depletion, and the high probability of error in this type of network, the SOSBRA proposed algorithm considers four criteria, battery level energy, mobility speed, number of hops and bandwidth for routing at the same time that can be very efficient and productive in routing.

## Supporting information

S1 Dataset (RAR)
